# Covariation of psychobiological stress regulation with valence and quantity of social interactions in everyday life: disentangling intra- and interindividual sources of variation

**DOI:** 10.1007/s00702-021-02359-3

**Published:** 2021-06-28

**Authors:** Martin Stoffel, Elvira Abbruzzese, Stefanie Rahn, Ulrike Bossmann, Markus Moessner, Beate Ditzen

**Affiliations:** 1 Institute of Medical Psychology, Center for Psychosocial Medicine, University Hospital, Heidelberg University, Bergheimer Straße 20, 69115 Heidelberg, Germany; 2 Clinical Psychology and Psychotherapy, Department of Psychology, University of Zurich, Zurich, Switzerland; 3 Institute of Psychosocial Prevention, Center for Psychotherapy Research, Center for Psychosocial Medicine, University Hospital Heidelberg, Bergheimer Straße 54, 69117 Heidelberg, Germany

**Keywords:** Social interactions, Social support, Stress buffering, Salivary cortisol, Salivary alpha-amylase, Psychobiological stress

## Abstract

While the overall effects of social relationships on stress and health have extensively been described, it remains unclear how the experience of social interactions covaries with the activity of psychobiological stress in everyday life. We hypothesized that the valence as well as quantitative characteristics of social interactions in everyday life would attenuate psychobiological stress. Sixty healthy participants provided data for the analyses. Using an ecological momentary assessment design, participants received 6 prompts on their smartphone for 4 days. At each prompt, they reported on social interactions since the last prompt (*any occurrence*, *frequency*, *duration*, *quality*, and *perceived social support*), current subjective stress, and provided one saliva sample for the analyses of cortisol (sCort) and alpha-amylase (sAA). Experiencing any contact within days as well as higher daily levels of contact quality and perceived social support were associated with reduced levels of sCort. Furthermore, on a daily level, experiencing at least one contact in-between prompts more often as well as having more contacts on average attenuated the sAA output. Perceived social support and contact quality as well as higher daily contact durations were associated with lower subjective stress. For sCort, daily levels of stress moderated the effects of experiencing any contact within days while daily perceived social support moderated the effects of subjective stress. For sAA, experiencing at least one contact in-between prompts more often on a daily level moderated the effects of subjective stress. There were no between-person effects throughout all analyses. The results show ecologically valid evidence for direct attenuating effects of social interactions on psychobiological stress as well as for the stress-buffering hypothesis in everyday life. Increasing the quantity and improving the valence of social interactions *on an intrapersonal level* can possibly reduce psychobiological stress and prevent its consequences.

**Supplementary Information:**

The online version contains supplementary material available at 10.1007/s00702-021-02359-3.

## Introduction

Social relationships are among the most important factors of physical and mental health (Holt-Lunstad et al. 2010; Umberson et al. 2010). As one important factor driving these effects, social integration (i.e., belonging to a social group and engaging in different social roles) is thought to have a direct positive impact (‘main effect’). For instance, being socially integrated can facilitate the access to important information, help to adhere to social norms (e.g., not taking illicit drugs) and health-behaviors (e.g., compliance to a medical treatment), and help oneself to define who he or she is (e.g., providing a sense of purpose, identity, and self-worth) (Cohen 2004). Beyond social integration, social interactions, defined as being in contact with at least one other person, can affect well-being to a great extent, depending on their valence and quantity: a recent meta-analysis showed that, during everyday life routines, negative and positive social interactions have an impact of negative and positive affect (Liu et al. 2019). In addition to social interactions, the effects of social support on health have extensively been investigated during the last decades. Social support can either be perceived or experienced while being classified as instrumental, emotional, appraisal, or informational (French et al. 2018). Therefore, feeling socially supported or receiving social support can be a result of social interactions, but can also be achieved through resources (e.g., financial resources), without any kind of direct interaction. Overall, the health-promoting effects of social support have been proven in large meta-analytic reviews (French et al. 2018; Heerde and Hemphill 2018). Correspondingly, social support has been suggested as one major mechanism by which social interactions can buffer the effects of feeling stressed, for instance by facilitating the reappraisal of stressful situations or by promoting the use of alternative coping behaviors (‘buffer effect’; Cohen 2004; Ditzen and Heinrichs 2014). Likewise, positive social interactions were shown to be associated with reduced stress (e.g., Bernstein et al. 2017). Consequently, positive social interactions and social support were shown to be of major relevance for buffering stress and its deleterious effects on mental and physical health (Dickman et al. 2020; Ditzen and Heinrichs 2014; Uchino and Way 2017), which have been well documented and replicated during the last few decades (Chrousos 2009; McEwen 2017).

To understand the neural underpinnings of these effects, neural correlates of social relationships and interactions have been described extensively, with results pointing to a complex interplay of central nervous system networks which have evolved during human evolution because they provided numerous advantages for survival (Porcelli et al. 2019). These ‘social brain’ structures mainly develop during early childhood and, because of the plasticity of the human brain, can be changed throughout adulthood (Atzil et al. 2018). Just like these social neural networks, stress systems have evolved to facilitate adaptivity and survival in the face of changing or demanding environments (Nesse et al. 2016). The most prominent systems are the ‘Hypothalamic-Pituitary-Adrenocortical Axis’ (HPA axis) and the ‘Sympathetic Adrenomedullary System’ (SAM axis) (Gunnar and Quevedo 2007; Ulrich-Lai and Herman 2009). The SAM axis is part of the sympathetic nervous system and is activated immediately after stressor exposure, its primary endpoints are catecholamines (epinephrine/norepinephrine) which are secreted from the adrenal medulla. As hormones, these catecholamines have a profound and systemic influence on the body, e.g., increasing heart rate or blood supply to the muscles. Given these various target locations, the SAM axis can be measured by various electrophysiological methods, but also in saliva by measuring the secretion of salivary alpha-amylase (sAA) (Nater et al. 2006). The HPA axis, on the other hand, is activated by the release of the corticotropin-releasing hormone from the hypothalamus. In turn, CRH leads to a secretion of ACTH from the pituitary into the blood stream, which acts to secrete corticosteroids, such as cortisol, from the adrenal gland. Just like catecholamines, these hormones have a variety of effects on the body, such as gluconeogenesis, changes in cardiovascular functioning, changes in the immune system, enhanced memory consolidation and many more (Sapolsky et al. 2000). Just like the SAM axis, the activity of the HPA axis can be measured in saliva, by quantifying levels of cortisol (sCort). Given these effects, it becomes clear that both stress axes help the body to be prepared for upcoming demands or challenges. Both stress axes are regulated by the same neural structures, such as parts of the prefrontal cortex (PFC), the anterior cingulate cortex (ACC), the amygdala, the bed nucleus of the stria terminalis, the hippocampus, the locus coeruleus, and the hypothalamus (Gunnar and Quevedo 2007). Interestingly, many of these structures were also identified to be of highest relevance for social interactions and social relations, especially the amygdala together with the PFC and the ACC (Bickart et al. 2014; Frith and Frith 2006; Von der Heide et al. 2014). Following this, converging evidence has shown that social buffering effects indeed work via an activation of the PFC as well as via a deactivation of the amygdala, of the ACC and of other frontal areas (Porcelli et al. 2019). In summary, there are numerous ways in which social interactions, social support or social relations (in general) can lead to altered neural activity patterns, which then were shown to exert inhibitory effects on central aspects of the human stress response (Hostinar et al. 2014; Uchino and Way 2017).

Given these associations, cross-sectional studies were conducted to investigate the effects of social interaction or social support on the activity of the HPA and the SAM axes following the exposure to a standardized laboratory stressor, the ‘Trier Social Stress Test’ (TSST) (see Ditzen and Heinrichs 2014 for a review). While these results clearly show support for the effects of social support and positive social interactions on the reactivity of the biological stress axes, studies which aimed at testing the underlying hypotheses outside the laboratory in people’s everyday lives yielded inconsistent results. These studies included the assessment of daily levels of ‘provision of prosocial behavior’ (Armstrong-Carter and Telzer 2021), daily positive or negative social interactions (Birditt et al. 2017), general levels of social integration (Dickman et al. 2020), general levels of social support by family and friends (Doane and Zeiders 2014), work social support (Evans and Steptoe 2001), social support as part of the psychosocial work environment (Evolahti et al. 2006), general levels of social support from a romantic partner (Giesbrecht et al. 2013), general levels of neighborhood social support (Karb et al. 2012), general levels of interpersonal social support (Luecken et al. 1997), general levels of social support (Rosal et al. 2004), daily levels of social support (Sayal et al. 2002), general levels of seeking social support as a coping style (Sladek et al. 2017), and ‘daily social connection scores’, composed of time spent interacting with others as well as of interaction quality (Sladek and Doane 2015). Of note, none of these studies investigated the within-person associations of social interactions and HPA- or SAM axis activation in everyday life, where they occur and exert their effects. Rather, social interactions were assessed via different psychometric scales or interviews at only one time point or, at a maximum, at one time at each day of the study. Using such retrospective questionnaires or interviews comes with various problems such as incomplete encoding and biased decoding of information (e.g., introduced by summarizing), heavy influences of current states (e.g., current feelings of loneliness in the evening) or further distortions introduced by contextual factors (e.g., being in a laboratory versus being in a crowded area with friends) (Bolger and Laurenceau 2013; Stone and Shiffman 2002). These systematic biases can heavily confound the results of such studies. Furthermore, such measurements are mainly used to measure between-person associations (e.g.: ‘Are general levels of social support associated with an altered activation of the stress axis?’) but not within-person variations (e.g.: ‘Are intrapersonal changes in social interactions in everyday life associated with an altered activation of the stress axes?’). However, past research has clearly shown the advantages of analyzing within-person variances when associations with sCort are to be assessed (Hruschka et al. 2005). Importantly, these problems might have occurred in many of the articles which reported on the effects of social relationships (social interactions, social integration, social support) on psychobiological stress systems in everyday life and this might explain the inconsistencies in the results reported thus far.

To capture within-person associations in addition to between-person associations, stress axes activity and momentary states need to be assessed simultaneously (or in close succession) in everyday life, e.g., using ecological momentary assessment techniques (EMA) (Shiffman et al. 2008). To our knowledge, only two studies assessed within-person associations of social interactions and sCort and/or sAA within days. In the first study by Bernstein et al. (2017), to quantify sCort levels within days, an EMA approach was used to assess six saliva samples on three consecutive days. On each occasion, participants were asked whether they were *currently* engaged in any social interaction and, if yes, how pleasant they rated the current interaction. Using this study design, the authors were able to distinguish momentary within-person effects of social interactions and their valence from between-person effects. The authors reported no significant effects of social interaction or its pleasantness on sCort levels. However, as the authors also discuss in their manuscript, coupling *current* states or experiences with *current* sCort levels comes with the limitation that changes in HPA axis activation can usually only be measured 10–20 min after exposure to altered states or environments (Kirschbaum et al. 1993). Therefore, the results of this study are only of limited use to interpret the effects of social interactions on HPA axis activation in everyday life. In the second study by Doerr et al. (2018), sCort was shown to covary between partners on a momentary level, which was more pronounced *without* partner interactions in-between prompts. Likewise, but only in women, there were covariations of sAA in couples which were independent of couple interactions in everyday life. These latter results suggest that being in a romantic relationship can have an impact on stress axes regulation and that, depending on social interactions between partners during everyday life routines, these covariations of sCort can be altered on a within-person level.

Overall, while the current state of research clearly implies that there could be covariations between social interactions, social support, and psychobiological stress, it remains unclear if this holds true in everyday life contexts. We aimed at closing this gap by investigating within- and between-person effects of social interactions on the activity of the HPA axis, as indicated by sCort, and the SAM axis, as indicated by sAA. Social interactions were subdivided by their quantitative features (duration, frequency, and occurrence) as well as by their valence (social support and contact quality). As form of social support, perceived social support as a result of social interactions was assessed. We hypothesized that (a) social interactions in everyday life could attenuate the activity of both stress axes and reduce subjective stress and (b) social interactions would interact with subjective stress to exert effects on both bodily stress systems (i.e., ‘stress buffering’).

## Methods

### Participants

Sixty-one overall healthy persons were recruited to participate in the study which was part of a larger research project (registered at the German Clinical Trials Register; DRKS00016846). In this study, all participants were recruited to either participate in an intervention designed to reduce stress or in a control group (group assignment; 0 = control group, 1 = intervention group). For the present analyses, we only use a subset of data generated in the assessments which can be used to interpret within- and between-participant associations of social interaction in daily life and sCort and sAA levels. Thus, we do not interpret possible effects of the intervention conducted in the study. One participant was excluded from all analyses since she did not provide any data besides those on demographics, leading to a total sample size of *N* = 60 (*n* = 31 in the intervention group). Participant characteristics are described in Table 1. Exclusion criteria were self-reported chronic serious physical diseases (e.g., neurological diseases), self-reported chronic psychiatric diseases (e.g., schizophrenic spectrum disorders), heavy smoking (≥ 20 cigarettes daily), current substance abuse or addictive disorders, and the permanent intake of psychotropic drugs. For inclusion, participants had to be employed adults (≥ 18 years old) and had to have access to a mobile internet device (e.g., smartphone) during their daily routines. Inclusion and exclusion criteria, together with demographic data were assessed via self-reports using a self-designed questionnaire. As demographic data, we assessed age (in years), sex (0 = male, 1 = female), body mass index (BMI; kg/m^2^), menstrual phase, coded as luteal or follicular (0 = no and 1 = yes; participants without a menstrual cycle were coded as 0 in both variables), and intake of hormonal contraceptives (0 = no, 1 = yes). In case of full participation, subjects received a monetary compensation of 40 Euros.

**Table 1:** Sample characteristics (*N* = 60) and descriptive data

	Mean (SD)^a^	Range	Minimum	Maximum
Age (years)	36.172 (11.611)	41	19	60
Body mass index	25.406 (4.138)	16.615	17.928	34.543
Minutes since wake up^b^	8.666 (10.693)	65.667	1	66.667
Sleep quality^c^	62.972 (12.248)	57.75	36	93.75
Intake of meal^d^	28.905 (7.455)	36.5	10.65	47.15
Intake of drink^d^	30.779 (7.694)	36.8	12.45	49.25
Physical activity^e^	30.377 (9.058)	47.022	13.278	60.3
Cigarettes smoked per day	2.379 (1.406)	5	1	6
Subjective stress^f^	36.521 (12.888)	56.87	2	58.87

	*n*^g^	%		
Sex (female)	20	33.333		
Hormonal contraception	8	13.333		
Follicular phase^h^	14	11.667		
Luteal phase^h^	10	8.333		
Intake of caffeine^i^	349	24.253		

^a^Mean and standard deviations (in brackets) across all measurement occasions and participants are reported

^b^Time awake (in minutes) before the first assessment of the day

^c^1 = very bad, 100 = very good

^d^0 = none, 100 = very much

^e^1 = sparsely active, 100 = very active

^f^1 = relaxed, 100 = stressed

^g^Frequencies for all measurement occasions and participants are reported

^h^Menstrual cycle phase was assessed before days one and two as well as before days three and four of the study

^i^Total number of occasions at which participants reported to have consumed caffeine since the last occasion. For further descriptions of the variables, please see section “Ecological momentary assessment”

### Ecological momentary assessment

To assess trajectories of sCort, sAA, subjective stress, and social interactions in daily life, we chose a combination of an event- and time-based sampling schedule (Shiffman et al. 2008) with six prompts per day. By doing so, diurnal variations in sCort and sAA (Miller et al. 2016; Nater et al. 2007) could be covered while reducing participant burden to a minimum (Hoyt et al. 2016). The schedule was implemented at 2 days at the beginning of the study (i.e., before the intervention). These assessments were then repeated 1 week thereafter (i.e., after the intervention) so that each participant could provide a maximum of 24 data points (i.e., self-reports and/or saliva samples) on 4 days (time in days; where days one and two = 0 and days three and four = 1).^1^ The first survey around the time of awakening (event; T1) triggered the following five sampling occasions, which took place 30 min (T2), 150 min (T3), 480 min (T4), and 720 min (T5) thereafter. The last measurement took place at bedtime (T6). At each prompt, participants received a text message with a link leading to the internet-based assessment. They were then asked to provide a saliva sample and subsequently to fill out several self-report questions using visual analogue scales (VAS) and dichotomous items. To control for state-variations in neuroendocrine stress levels (sAA and sCort), several control variables were assessed (Adam and Kumari 2009; Strahler et al. 2017). Among them, at T2 to T6, food and drink intake (0 = none, 100 = very much), number of cigarettes smoked, the amount of physical activity (1 = sparsely active, 100 = very active), and caffeine intake (0 = no, 1 = yes) since the last saliva sample. In addition, at T1, participants rated the quality of their sleep during the past night on a VAS (1 = very bad, 100 = very good) and indicated the time they were awake (in minutes) before the first assessment of the day (minutes since awakening). Adherence to the sampling protocol was assured in accordance with standard procedures (Adam and Kumari 2009). On average, participants provided 95.13% (SD = 9.4%) of the 24 saliva samples and completed 94.93% (SD = 9.4%) of the 24 questionnaires. Furthermore, on average, 92.3% (SD = 12.9%) valid saliva samples and questionnaires were provided together.

#### Assessment of social interactions and subjective stress in daily life

At T2–T6, social interactions in daily life were assessed by asking participants whether they had any kind of social interaction since the last prompt or, only at T1, during the previous night (*contact*; 0 = no, 1 = yes). At T2–T6, if participants reported to have interacted in-between prompts, they were subsequently asked how many contacts they experienced (*contact frequency*; 1 to > 6), how long they felt that each contact lasted (*contact duration*; VAS; 1 = short, 100 = long), how they rated each interaction (*contact quality*; VAS; 1 = negative, 100 = positive) and to what extent they felt socially supported (*perceived social support*; VAS; 1 = sparsely, 100 = much). Since participants could report on several contacts between prompts and, thus, there could be more than one rating on all variables (except *contact frequency*), mean values for each variable, which represent the average rating in-between two prompts, were calculated.^2^ Subjective stress was assessed at each prompt (T1–T6) by asking participants ‘How do you feel at the moment?’ (VAS; 1 = relaxed, 100 = stressed).

### Salivary cortisol and salivary alpha-amylase

Saliva samples were collected with SaliCab^®^ tubes (RE69985, IBL, Hamburg, Germany). At each prompt (T1–T6), participants were instructed to passively drool the saliva through a plastic straw into the vial for 1 min. At the end of each day, participants stored the saliva samples in their refrigerators. The samples were then returned to the laboratory immediately on the day after the first 2 days and the last 2 days (1 week later) of the study. The samples were stored at − 80 °C until analyses for no more than 6 months. Salivary cortisol was analyzed using a commercially available enzyme-linked immunosorbent assay (RE52611; IBL, Hamburg, Germany) following the manufacturers’ instructions. Alpha-amylase was analyzed using a kinetic colorimetric kit with reagents from Roche (Roche Diagnostics, Mannheim, Germany). Biological data were generated in the stress biomarkers lab at the Institute of Medical Psychology, Heidelberg. All analyses were conducted in duplicates and mean values were used for all analyses. The intra-assay coefficient of variation (CV) was 5.44% for sCort and 3.87% for sAA. The inter-assay CV was 6.49% for sAA and 6.96% for sCort.

### Statistical analyses

Data preparation and statistical analyses were performed in the statistical environment R (R Core Team 2020). Given the nested structure of the data, as measurements on level 1 (L1) were nested in days on level 2 (L2) which were nested in participants on level 3 (L3), multilevel models (MLM) were used to test the main hypotheses.

### Centering of focal predictors

To disentangle the effects of the focal predictors (*contact, contact frequency*, *contact duration, contact quality,* and *perceived social support)* within and between participants, centering strategies for three-level MLM were used (see Brincks et al. 2017). These strategies aimed at decomposing the variances into three components: (1) within-person within days (momentary level) on L1, (2) within-person from day to day on L2 (day-level) and (3) between-person (person-level) on L3. To obtain the components on L1, focal predictors on L1 were centered on person-specific daily means for the respective predictors. In a next step, the person-specific daily means on L2 were centered on the person-specific means to obtain the components on L2. In a last step, to obtain the between-person components on L3, each person-specific mean was centered on the grand mean (i.e., the mean of all person-means). Following this approach, the three components of the focal predictors can provide substantially different information:

L1 components on the momentary level (within-person, within days): *Is the momentary concentration of sCort/sAA or the subjective stress lower when the person experienced higher levels of the focal predictor in-between prompts than usual on a specific day?*

L2 components on the day-level (within-person, across days): *Is the concentration of sCort/sAA or the subjective stress lower when the person experiences greater averaged daily levels of the focal predictor than he/she experiences on average (e.g., compared to the other days of the study on which average levels of the focal predictor are reported by the person)?*

L3 components on the person-level (between-person): *Is the concentration of sCort/sAA or the subjective stress lower for a person who experiences greater average levels of the focal predictor than other persons?*

### General approach

The MLMs were fitted with the ‘lme’ function of the ‘nlme’ package (Pinheiro et al. 2020) with a restricted maximum likelihood method of estimation (REML). Before analyses, the distributional properties of sCort, sAA, and subjective stress were investigated. Since sAA and sCort were found to be positively skewed, both were transformed to the natural logarithm to help with the normality of the MLM residuals. Thereafter, outliers beyond three SDs of means of all dependent variables were excluded (Adam and Kumari 2009). In a next step, together with the values on subjective stress, the logarithmized sCort and sAA values were plotted to identify nonlinear time trends. Following this, linear and quadratic time trends within days were added as fixed effects to detrend the time series for all three parameters. Random effects of time within days were added on L2 or L3 to test their ability to improve the model fit. These steps were performed by fitting new models (each including one random effect) and by comparing them to their predecessors (e.g., random-intercept only models) using likelihood-ratio tests as well as the Bayesian information criterion (BIC). Distributional assumptions for the model residuals were tested according to standard procedures (Pinheiro and Bates 2000). After finding the best fitting baseline model, separate MLMs were fitted, each including the L1, the L2 und the L3 components of one focal predictor to test the hypotheses. To avoid erroneous estimates for relevant fixed effects, all L1 and L2 components of the focal predictors were tested as random effects on L2 and/or L3 as described above (Baird and Maxwell 2016). In addition, in all models, we also chose to control for group assignment (L3), the effects of time in days (L2) as well as of their interaction. This step was performed to control for a potential intervention effect.^3^ All covariates, except time within days (uncentered), group, and time in days (see section “Ecological momentary assessment”), were centered on their grand mean. Results of covariates and variances of random effects are not reported since they are not at focus of the hypotheses. Beyond this general approach, outcome-dependent modeling choices (e.g., specific covariates or the random effects structure of the final models) are described separately for each outcome in the following two paragraphs.

### Salivary cortisol and alpha-amylase: testing direct effects of social interactions

In these models, food, drink, and cigarette consumption as well as physical activity were added as covariates on L1. On L2, sleeping quality, minutes since awakening and menstrual phase were added. Finally, the person-level covariates sex, age, and the intake of hormonal contraception were added on L3. Since it is known that neuroendocrine outcomes in everyday life show high levels of variability across days and between participants (e.g., Almeida et al. 2009; Segerstrom et al. 2017), we chose to test the linear and quadratic trends within days as random effects on L2 and L3. For both, sAA and sCort, the best model fit was achieved by setting the linear and quadratic trends of time within days as random on L3. All distributional assumptions were met.

### Subjective stress: testing direct effects of social interactions

Sex and age were added as covariates on L3. Furthermore, random effects of linear and quadratic time trends on L2 and L3 were shown to further significantly improve the model fit and, thus, were kept for the final model. In these models, the residuals on L1 were found to be not independent. Consequently, a continuous autoregressive correlation structure of order 1 (Pinheiro and Bates 2000) as a function of time between two adjacent prompts was added to the baseline model and significantly improved its fit. All further distributional assumptions were met.

### Moderation analyses: testing the stress-buffering hypothesis

Moderation analyses were conducted to test whether significant associations of the focal predictors with the stress axes (‘direct effects’) would be moderated by subjective stress. To decompose the variance of subjective stress into three components (L1–L3), it was centered as described above. The analyses were performed by refitting the MLMs used to generate the significant main results, with the only difference that, instead of main effects, statistical interactions of subjective stress (on L1–L3) with the significant focal predictors (on L1, L2 or L3) were analyzed. Separate models were fitted to test each interaction (e.g., an interaction of contact quality on L2 with subjective stress on L1, L2, and L3 in three separate MLMs). In case of cross-level interactions, the predictor on the higher level was interpreted as moderator (Andersson et al. 2014). Interaction plots were built using the R packages ‘effects’ (Fox and Weisberg 2019) and ‘ggplot2’ (Wickham 2016).

### Sensitivity analyses

To further ensure that the intervention did not alter the effects of our focal predictors on the psychobiological indicators of stress, sensitivity analyses were carried out for all significant results from the main analyses. All corresponding models were refitted while including three-way interactions of group, time (pre/post) and the effect we found in our analyses (e.g., of contact quality).

## Results

Descriptive data pertaining to all control variables and subjective stress are presented in Table 1 while descriptive information for all items used to assess social interactions are reported in Table 2. The *direct* effects (estimates, *p* values, and standard errors) of the focal predictors on sCort, sAA, and subjective stress are presented in Table 3. In the following, the effects are described for each focal predictor separately.

**Table 2:** Descriptive data for items assessing social interactions in everyday life

	Mean (SD)^a^	Range	Minimum	Maximum
Contact quality^b^	73.373 (9.627)	45.818	54.182	100
Perceived social support^c^	67.412 (11.492)	52.896	37.667	90.563
Contact frequency^d^	2.648 (1.128)	5.85	0	5.85
Contact duration^e^	50.306 (12.972)	66.46	20.798	87.258

	*n*^f^	%		
Contact^g^	839	58.304		

^a^Mean and standard deviations (in brackets) across all measurement occasions and participants are reported

^b^1 = negative, 100 = positive

^c^1 = sparsely, 100 = much

^d^Average number of contacts, from 1 to > 6 contacts

^e^1 = short, 100 = long

^f^Frequencies for all measurement occasions and participants are reported

^g^Total number of occasions at which participants reported to have had at least one contact since the last occasion

**Table 3:** Summary of main results

Focal predictor	Fixed effects					
	sCort (logarithmized)		sAA (logarithmized)		Subjective stress	
	Estimates (SE)	*p*	Estimates (SE)	*p*	Estimates (SE)	*p*
Contact (within days)	− 0.128 (0.050)	0.011*	0.017 (0.057)	0.759	0.896 (1.202)	0.456
Contact (across days)	− 0.097 (0.109)	0.373	− 0.376 (0.137)	0.007*	− 1.772 (4.259)	0.678
Contact (between participants)	− 0.371 (0.266)	0.170	0.288 (0.590)	0.628	− 11.756 (0.166)	0.166
Contact frequency (within days)	− 0.008 (0.009)	0.385	− 0.011 (0.011)	0.311	0.311 (0.243)	0.200
Contact frequency (across days)	0.022 (0.018)	0.225	− 0.074 (0.022)	0.001*	0.982 (0.697)	0.160
Contact frequency (between participants)	− 0.055 (0.039)	0.167	0.039 (0.088)	0.664	− 1.110 (1.394)	0.429
Contact quality (within days)	− 0.001 (0.001)	0.384	− 0.003 (0.002)	0.070	− 0.293 (0.040)	0.000*
Contact quality (across days)	− 0.006 (0.002)	0.013*	0.002 (0.003)	0.488	− 0.538 (0.079)	0.000*
Contact quality (between participants)	0.001 (0.005)	0.821	0.019 (0.010)	0.061	− 0.268 (0.174)	0.129
Perceived social support (within days)	0.001 (0.001)	0.558	0.000 (0.001)	0.971	− 0.130 (0.032)	0.000*
Perceived social support (across days)	− 0.005 (0.002)	0.025*	0.000 (0.003)	0.999	− 0.322 (0.075)	0.000*
Perceived social support (between participants)	− 0.003 (0.004)	0.500	0.008 (0.008)	0.293	− 0.094 (0.141)	0.508
Contact duration (within days)	0.000 (0.001)	0.689	0.001 (0.001)	0.519	− 0.051 (0.030)	0.089
Contact duration (across days)	− 0.003 (0.002)	0.086	− 0.000 (0.002)	0.892	− 0.153 (0.064)	0.017*
Contact duration (between participants)	− 0.003 (0.004)	0.359	− 0.003 (0.007)	0.729	0.166 (0.137)	0.230

Table depicts point estimates (standard errors for fixed effects in brackets) from multilevel models. For centering strategies, please see section “Statistical analyses”. Random effects and control variables are not reported because they are not of interest for the research question

*p < 0.05

### Direct effects of social interactions

#### Contact

Experiencing at least one social interaction in-between two prompts (contact) was associated with reduced sCort levels on a momentary level, especially if that interaction was rather the exception than the rule on that given day (i.e., when the participant reported to have experienced at least one social interaction in-between prompts less frequent on average on this day). In other words: there was a significant within-person effect of social interaction on sCort on L1. To the contrary, experiencing at least one social interaction in-between prompts *more often on average*, either on one day compared to the average across all days (within-person; L2) or across all days compared to the average of all persons (between-person; L3), was not associated with reduced levels of sCort. For sAA, experiencing at least one social contact in-between two prompts more frequent on average on one of the EMA days, compared to the person-specific average across days, was associated with lower average levels of sAA in everyday life on L2. However, these effects could not be found on L1 or L3. Likewise, the variable was not associated with within-person or between-person differences in subjective stress on either of the three levels.

#### Contact frequency

Contact frequency showed a negative effect on sAA concentrations on L2, thereby indicating that a higher total number of contacts on one day, compared to days with lower levels of contact frequency, lead to lower levels of sAA. On the other hand, within-day variations (L1) or between-subject differences (L3) in contact frequency showed no effect on sAA. Regarding sCort and subjective stress, contact frequency was not of relevance on any of the three levels.

#### Contact duration

Contact duration did not show any effect on either sAA or sCort (with exception of a non-significant trend on L2). However, higher levels of daily contact duration were associated with lower levels of subjective stress on L2. Furthermore, on L1, there was a non-significant trend for an association of longer contact duration since the last prompt and momentary lower levels of subjective stress. There were no between-person effects of contact duration on subjective stress (L3).

#### Perceived social support

Feeling more socially supported on average on a given day was found to be associated with lower average sCort levels on that day (L2). However, there were no effects of perceived social support on L1 or L3 for sCort and none for sAA. In addition, higher levels of perceived social support within days (L1) and across days (L2) were found to be associated with lower levels of subjective stress. There were no between-person effects of perceived social support on subjective stress (L3).

#### Contact quality

For sCort, higher average daily levels of contact quality were associated with attenuated average concentration levels on the day-level (L2) while there were no effects on L1 or L3. For sAA, there were no significant effects on either of the three levels. However, there were trends for effects of contact quality on L1 and L3, which failed to reach significance. Subjective stress was found to be negatively associated with contact quality on L1 and L2, but not on L3 (between-person).

#### Results of sensitivity analyses

The results of the sensitivity analyses revealed no significant three-way interactions of group, time, and the focal predictors of relevance (i.e., which showed significant direct effects on the outcomes, see above), thereby indicating that the significant effects were not dependent on whether participants took part in the intervention or not.

### Interaction effects of social interaction and stress on stress axes regulation

**Fig. 1:**
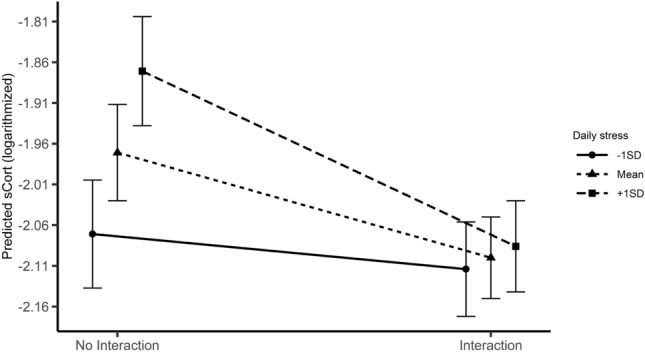
Average predicted values of sCort (logarithmized) as a function of the interaction of having any contact in-between prompts (L1) and daily levels of stress (L2). To facilitate interpretability, predicted values and standard errors were estimated only for values > 0 and < 0 of the centered variable ‘contact’. They were then averaged for all cases where any contact occurred in-between prompts (values > 0; ‘Interaction’) and for all cases without contact in-between prompts (values < 0; ‘No interaction’). Mean values as well as one standard deviation below and above the mean were used as grouping levels for the moderator. The error bars indicate standard errors

**Fig. 2:**
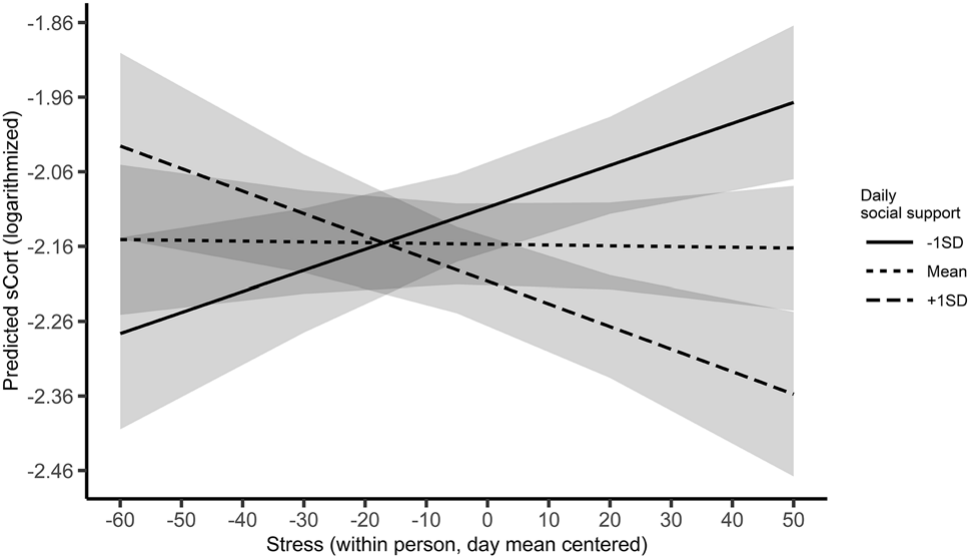
Average predicted sCort (logarithmized) as a function of the cross-over interaction of subjective stress (L1) and daily levels of social support (L2). Mean values as well as one standard deviation below and above the mean were used as grouping levels for the moderator. The ribbons indicate standard errors

**Fig. 3:**
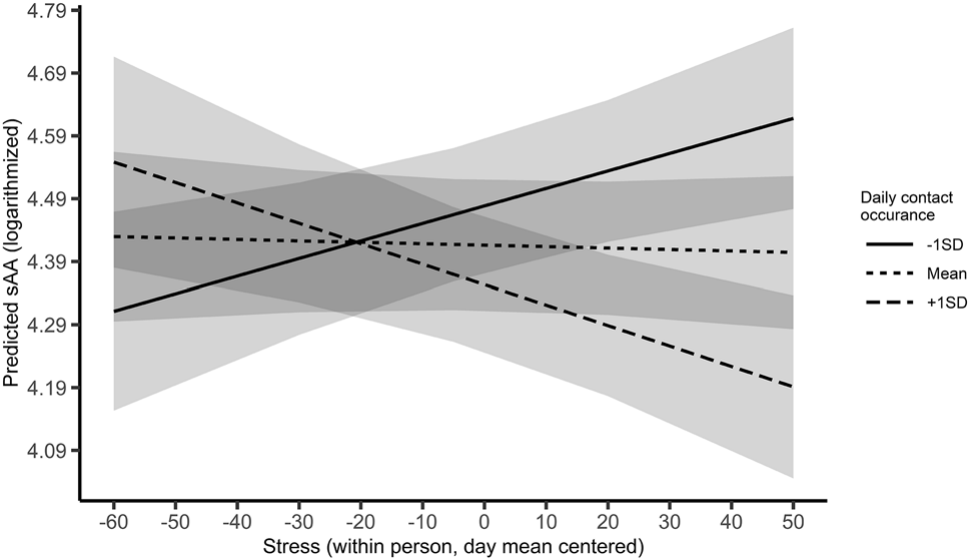
Average predicted sAA (logarithmized) as a function of the cross-over interaction effect of subjective stress (L1) and daily levels of contact occurrences (L2). Mean values as well as one standard deviation below and above the mean were used as grouping levels for the moderator. The ribbons indicate standard errors

The results showed a cross-level interaction of contact (L1) and daily stress (L2) on sCort levels (*b* = − 0.009, SE = 0.005, *p* = 0.040; see Fig. 1). When daily stress was high (as compared to other days within a person), having at least one contact in-between two prompts attenuated the secretion of sCort to a level comparable to low-stress days. In turn, without at least one contact in-between prompts, high levels of daily stress were associated with high levels of sCort. Correspondingly, in the interaction model, there were main effects for daily levels of stress (L2; *b* = 0.006, SE = 0.002, *p* = 0.003) and having any social contact since the last prompt (contact) (L1; *b* = − 0.129, SE = 0.050, *p* = 0.010). The next model revealed a cross-level interaction between daily levels of perceived social support (L2) and momentary subjective stress (L1) on sCort levels (*b* = − 0.0003, SE = 0.0001, *p* = 0.020; see Fig. 2). Although there was no overall effect of momentary subjective stress (L1; *p* > 0.05) on sCort, the interaction clearly shows that the effect of stress is opposite, depending on the daily levels of perceived social support (L2). Thus, the interaction is not only cross-level but also cross-over. The main effect of perceived social support on L2 remained significant (*b* = − 0.005, SE = 0.002, *p* = 0.019). For sAA, there was a similar cross-level interaction between contact on L2 (i.e., experiencing at least one social contact in-between two prompts more frequent on average on one of the EMA days, compared to the other days) and momentary subjective stress on L1 (*b* = − 0.019, SE = 0.009, *p* = 0.045; see Fig. 3). This cross-over interaction indicates that, despite there is no overall effect of momentary subjective stress on L1 (*p* > 0.05), the effect of stress on sAA is reversed, depending on the daily levels of contact occurrences (i.e., experiencing at least one contact in-between prompts more often on average on a given day). The main effect of contact on L2 remained significant in this model (*b* = − 0.391, SE = 0.138, *p* = 0.005). There were no further significant interactions between subjective stress and the focal predictors of relevance. For illustrative purposes, all graphs depicting interaction effects were also created using untransformed sCort and sAA values. They can be found in the Supplementary Information.

## Discussion

The results of the study suggest that social interactions can have immediate and prolonged direct attenuating effects on healthy participants’ biological stress systems and on the subjective feeling of stress in everyday life. Furthermore, the effects presented in this study show support for the stress-buffering hypothesis (Ditzen and Heinrichs 2014), because subjective stress and social interactions in everyday life were shown to interact with regard to the activity of the stress axes. Of note, all results were found only on an intrapersonal level and there were no between-person effects of social interaction.

Regarding the stress-buffer hypothesis, the results indicated that having *any contact* on high-stress days can attenuate the otherwise increased output of the HPA axis. Furthermore, experiencing higher daily levels of perceived social support inversed the otherwise positive association of stress and HPA axis activation. Likewise, when at least one contact in-between two prompts occured more often on one day, as compared to how often a person reported to have experienced at least one contact in-between prompts on average, the positive association of stress with the activity of the SAM axis seemed to be inverted. Both cross-over interactions suggest that subjective stress can be either (a) not of relevance for stress axes activation, when social parameters are at a usual daily level within a person, (b) positively associated with stress axes activation, when social parameters are lower than usual, or even (c) negatively associated with the secretion of sCort and sAA in daily life, when social parameters are higher than usual. Thus, experiencing high levels of stress can possibly even be associated with *reduced* levels of stress axes activation, given that relevant social parameters are considerably higher than usual for a person on a given day. This suggest that engaging in social interactions (especially considerably more often or when of higher quality than usual) requires effort and resources, which could lead to subjective ratings of being ‘stressed’ rather than ‘relaxed’. Interestingly, these results are in line with a recent study in which the frequency of social contacts in everyday life was clearly associated with higher levels of stress (Gloster et al. 2020). Accordingly, such results had previously been reported and explained by others (Gleason et al. 2008), with the most recent evidence pointing towards a role of social support visibility: when social support is experienced as such, it might be accompanied by *negative* effects on psychological outcomes (Zee and Bolger 2019). However, given the attenuating *direct effects* of the social parameters involved on the biological stress axes, it seems reasonable that increased subjective stress can be accompanied by a reduced output of the stress axes—but only if ‘stress’ is experienced in positive social contexts. Conversely, being subjectively stressed and socially deprived at the same time can lead to a markedly increased stress axes output.

Consequently, beyond these interactions, the direct effects of social interaction on the stress axes seem to be of high importance: for sCort, experiencing any social interaction (contact) in everyday life was associated with lower concentrations on a momentary level. There were, however, no other effects on this level, thereby indicating that neither momentary levels of contact quality or perceived social support nor the momentary number or the duration of social interactions matter when explaining why social interactions buffer levels of sCort within days. However, higher levels of average daily perceived social support and contact quality were associated with attenuated average levels of sCort on the respective day. This implicates that changes in these parameters in everyday life are not of immediate (momentary) relevance but do exert their effects by reducing the average daily output of sCort. For sAA, effects were only of relevance on the day-level: having at least one contact in-between prompts more often as well as having more contacts on average (contact frequency) attenuated the average daily sAA output. However, there were no effects of other aspects of social interaction, which indicates that the valence of contacts does not matter for sAA (i.e., contact quality and perceived social support). In turn, subjective stress was mainly shown to be attenuated by within-person variations in contact valence. As such, experiencing higher levels of contact quality and perceived social support within and across days (both within-person) were associated with lower levels of subjective stress. In addition, experiencing higher levels of daily contact duration, but none of the other quantitative characteristics (contact, contact frequency), were associated with lower subjective stress.

Taken together, this pattern of direct effects and interaction effects suggests that there are likely other important specific nuances of contact quality^4^ or perceived social support (e.g., Siewert et al. 2011), or even entirely different characteristics (e.g., Bernstein et al. 2017), and that these factors could be of relevance for an in-depth understanding of the quantitative effects of social interactions. Another important factor could be to consider person-level moderators. For instance, a recent study by Han et al. (2021) found that romantic attachment style moderated the effects of partner presence on electrodermal activity (as an indicator of the autonomic nervous system) in everyday life. Following this, it seems also noteworthy that, beside valence and quantity, it could be of highest relevance to consider with whom the contact was made (e.g., romantic partner vs. stranger) and what kind of relationship the persons were engaged in (e.g., romantic vs. competitive). Correspondingly, there is evidence showing specific influences of leadership (Herr et al. 2019), friendship (Keneski et al. 2017) or couple relationships (Doerr et al. 2018) on sCort in everyday life. In addition, contact often happens in groups, where different characters interact based on a variety of conditions, such as social roles, which themselves could be a measure of social integration (Dickman et al. 2020). In summary, this elucidates the complexity of the field and highlights challenges for future research because, to disentangle these mechanisms, large sample sizes and an even larger number of sampling occasions are needed. Yet, the present study helps to explain some of these mechanisms and adds to the broad body of evidence in which measures of social contact had been assessed as standalone person-level or retrospectively assessed day-level variable (Armstrong-Carter and Telzer 2021; Birditt et al. 2017; Dickman et al. 2020; Doane and Zeiders 2014; Evans and Steptoe 2001; Evolahti et al. 2006; Giesbrecht et al. 2013; Karb et al. 2012; Luecken et al. 1997; Rosal et al. 2004; Sayal et al. 2002; Sladek and Doane 2015; Sladek et al. 2017) by showing that quantitative characteristics and the valence of social interactions can attenuate biological and subjective stress in real-time during everyday life routines, but also on average across days. Importantly, they also provide scarce ecologically valid evidence for a stress-buffering role of social interactions on biological stress axes.^5^ Finally, the results of the present study are an indirect validation of the theories which aimed at describing the neural mechanisms on how social interactions buffer the activity of the bodily stress axes (Hostinar et al. 2014; Uchino and Way 2017).

The study has several limitations which need to be considered. First, the data analyzed here were derived from an interventional study and, thus, one part of the sample did receive an intervention focused at reducing stress. This could have an impact on the associations of social interactions and stress. Because of this, we chose to control for group assignment and time in days (i.e., pre- and post-intervention) as well as their interaction in all analyses. Controlling for this group-by-time interaction directly and in additional sensitivity analyses (i.e., a possible intervention effect) did not have an impact on the results reported here. However, given that a potential impact of the intervention cannot be ruled out in its entirety and because analyses with only pre-intervention data were hindered by a reduced statistical power as well as by overparameterization of the statistical models, further studies are needed to replicate the present results. Second, the sample size of 60 participants is only moderate, and a total of six samples on 4 days could result in imprecise estimates for person- or day-means (which are needed to perform within-person centering strategies). Third, participants in the study were all healthy and employed and, thus, the generalizability to other populations (such as clinical populations) is limited. Correspondingly, there was an unequal sex distribution in the sample, with about two-thirds of the sample being male and only one-third female. Sex differences in response to stress in general are well known (Hodes and Epperson 2019). Regarding psychobiological stress, while self-reported stress is higher in women, sCort reactivity to stress was shown to be lower in women then in men, mainly because of differences in circulating sex hormones (Juster et al. 2016; Kirschbaum et al. 1999). A less clear picture can be drawn for sAA, where (a) men were shown to secrete either higher (van Stegeren et al. 2008) or equal (Nater et al. 2007) baseline levels, (b) reactivity to stress was shown to be independent from sex (e.g., Maruyama et al. 2012), higher for men than for women (Smeets 2010) or vice versa (Carr et al. 2016), and where (c) stress reactivity in women was shown to be higher either in the luteal (Espin et al. 2019) or in the follicular phase (Hlavacova et al. 2017). Beyond these effects, previous research has also shown that the proposed stress-attenuating effects of social interactions can be sex-specific, with men seemingly benefiting more from social support (Ditzen and Heinrichs 2014; Kirschbaum et al. 1995). Given this overall picture, we decided to not only control for age and sex, but also menstrual cycle phase and intake of hormonal contraceptives. While this might cover a lot of variance introduced by biological sex differences, it would have been ideal to assess sex hormones directly (Juster et al. 2016), which was hindered by monetary restrictions, or to recruit a more balanced sample. Thus, there still might be variance introduced by sex differences which could not be accounted for. Fourth, given that coupling current levels of sCort with current social interactions would lead to erroneous estimates of their association (i.e., because changes in sCort occur only 15–20 min after exposure to a stimulus; see Bernstein et al. 2017), we decided to ask for the social interactions which had happened in-between prompts. However, this comes with the limitation that participants had to recall these information and memory distortions could appear. However, the timeframes in-between prompts were relatively short and, thus, it is unlikely that the assessed information on social contacts were heavily distorted. As alternatives, it would have been necessary to either allow user-initiated prompts (i.e., whenever there currently is a social interaction) or to assess social interactions much more frequently (e.g., every 30 min). However, both approaches did not seem viable because they would have come with increased participant burden and possibly a reduced compliance (Williams et al. 2021). In addition, it would not be feasible for participants to report on their interactions during their everyday life routines in such a high frequency and, most importantly, the reporting itself could have interfered with social interactions (e.g., by interrupting conversations), thereby reducing ecological validity.

In conclusion, the results show evidence for direct attenuating effects of social interactions on psychobiological stress as well as for the stress-buffering hypothesis. Of note, these results were only found on an intrapersonal level. In contrast, between-person effects of social interaction, such as having more social contacts on average (across all persons) or experiencing more social support as compared to others, were of no relevance. Therefore, increasing the quantity and improving the valence of social contacts—*in comparison to the quantity and valence experienced routinely—*can possibly reduce psychobiological stress and prevent its consequences. Taken together, these results are adding to the growing body of literature on how active social relationships alleviate the burden of stress and increase mental and physical health.

## Supplementary Information

Below is the link to the electronic supplementary material.

Supplementary file1 (DOCX 608 kb)

## Data Availability

The data that support the findings of this study are available from the corresponding author, MS, upon reasonable request.

## Footnotes

1 Of note, given that the menstrual cycle phase might change within 1 week, it was assessed prior to the first and, again, prior to the last 2 days of the assessments.

2 For example, when a participant reported on two contacts between the third and fourth prompt and rated one with a VAS rating of 50 and the other one with a VAS rating of 100, the mean contact quality since the third prompt would be at 75 points on the VAS.

3 Using only pre-intervention data—and thereby completely eliminating a possible confounding by the intervention—was not feasible with the present dataset: given the high number of parameters to be estimated together with the reduced data set, overparameterization would occur, leading to models with limited interpretability, even if convergence would be possible (Bates et al. 2015). However, simplifying the structure of the models was not an option because an appropriate detrending of the time-series (e.g., considering quadratic trends) would still have been necessary (see Hruschka et al. 2005 for details; Wang and Maxwell 2015), as would have controlling for a wide variety of control variables known to influence sCort and sAA (Strahler et al. 2017). Likewise, further parameters as those needed to control for interindividual differences in the effects of the predictors (e.g., random slopes for the change of sCort or sAA within days) or possibly those controlling for dependencies in the residuals (e.g., autocorrelation) would need to be considered. In addition to leading to an inappropriate model structure, such simplifications would play a part in reducing the statistical power, which already would have been reduced (e.g., given an increased error variance), mainly because half of the assessments would be missing because of truncating the data (see Lane and Hennes 2018 for more details). This would result in an increased probability of committing type II errors (as compared to the models in which all available data were used). In addition, the person- and day-level means of the focal predictors, used to decompose the variance into within- and between-person components, would have a reduced reliability, given that they would only be composed of between 2 (L2) or 12 (L1) measurements (Wang and Maxwell 2015). Lastly, the loss of observations would lead to a reduced ecological validity. In summary, the research questions could not have been investigated with a reduced dataset and, furthermore, comparisons with results from simplified sensitivity analyses, in which only pre-intervention data were used, to the main analyses would not be meaningful.

4 However, it is noteworthy that contact valence was assessed on visual analogue scales and, consequently, negative social interactions, which can have a negative impact on psychobiological stress (e.g., Friedman et al. 2012), could also be assessed and were part of the analyses.

5 However, previous studies showed that levels of perceived social support or being engaged in any social interaction during daily life routines can moderate the effects of momentary rumination and depression on heart rate variability (HRV; as another marker associated with the SAM axis) (Gerteis and Schwerdtfeger 2016; Schwerdtfeger and Friedrich-Mai 2009). Given the supposed association of sAA and HRV, these results could be in line with those described in the present work.
