# Supplementary material for: Covariation of psychobiological stress regulation with valence and quantity of social interactions in everyday life: disentangling intra- and interindividual sources of variation
Source: J Neural Transm (Vienna). 2021 Jun 28;128(9):1381–95. doi: 10.1007/s00702-021-02359-3 (PMC8423684; doi:10.1007/s00702-021-02359-3)
Supplement: Supplementary file 1 — Supplementary file1 (DOCX 608 kb) [file 702_2021_2359_MOESM1_ESM.docx]

# Supplementary Information to:

# Covariation of psychobiological stress regulation with valence and quantity of social interactions in everyday life: disentangling intra- and interindividual sources of variation.

Martin Stoffel^a*^, Elvira Abbruzzese^b^, Stefanie Rahn^a^, Ulrike Bossmann^a^, Markus Moessner^c^ & Beate Ditzen^a*^

^a^Institute of Medical Psychology, Center for Psychosocial Medicine, University Hospital, Heidelberg University, Bergheimer Straße 20, 69115 Heidelberg, Germany

^b^Clinical Psychology and Psychotherapy, Department of Psychology, University of Zurich, Zurich, Switzerland

^c^Institute of Psychosocial Prevention, Center for Psychotherapy Research, Center for Psychosocial Medicine, University Hospital Heidelberg, Bergheimer Straße 54, D-69117 Heidelberg, Germany

**Corresponding authors**

*Martin Stoffel, Institute of Medical Psychology, Center for Psychosocial Medicine, University Hospital, Heidelberg University, Bergheimer Str. 20, 69115 Heidelberg, Germany, Phone: +49 6221 56 8436, Fax: +49 6221 56 5303, E-Mail: [martin.stoffel@med.uni-heidelberg.de](mailto:martin.stoffel@med.uni-heidelberg.de); ORCID-ID: 0000-0002-8935-0853

*Beate Ditzen, Institute of Medical Psychology, Center for Psychosocial Medicine, University Hospital, Heidelberg University, Bergheimer Str. 20, 69115 Heidelberg, Germany, Phone: +49 6221 56 8150, Fax: +49 6221 56 5303, E-Mail: [beate.ditzen@med.uni-heidelberg.de](mailto:beate.ditzen@med.uni-heidelberg.de); ORCID-ID: 0000-0001-5853-457


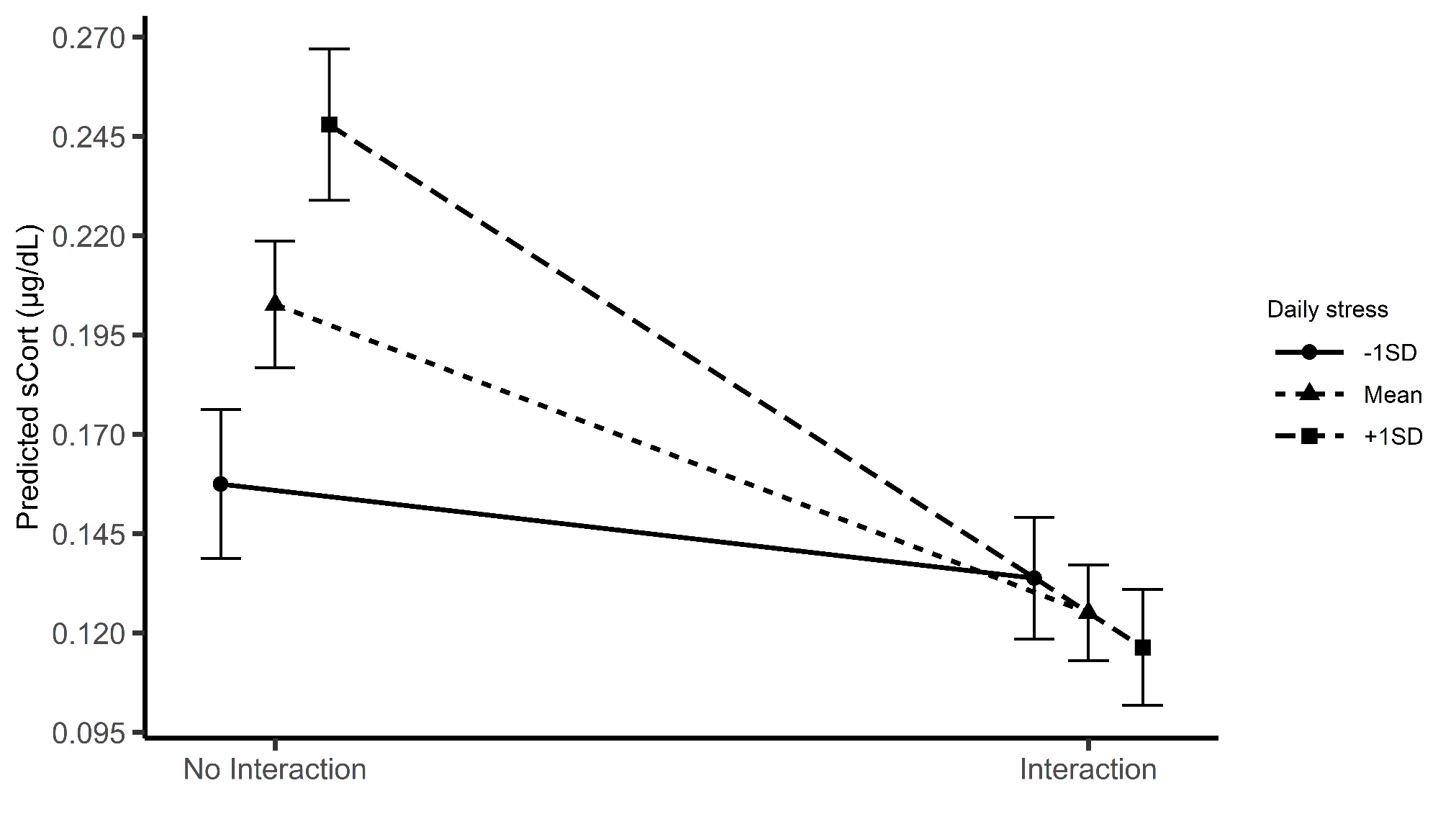
**Fig. S1 Average predicted values of sCort as a function of the interaction of having any contact in-between prompts (L1) and daily levels of stress (L2). To facilitate interpretability, predicted values and standard errors were estimated only for values > 0 and < 0 of the centered variable ‘contact’. They were then averaged for all cases where any contact occurred in between prompts (values > 0; ‘Interaction’) and for all cases without contact in between prompts (values < 0; ‘No interaction’). Mean values as well as one standard deviation below and above the mean were used as grouping levels for the moderator. The error bars indicate standard errors. Given that untransformed sCort values were used, the figure does not depict the fixed effects described in the main text.**

**
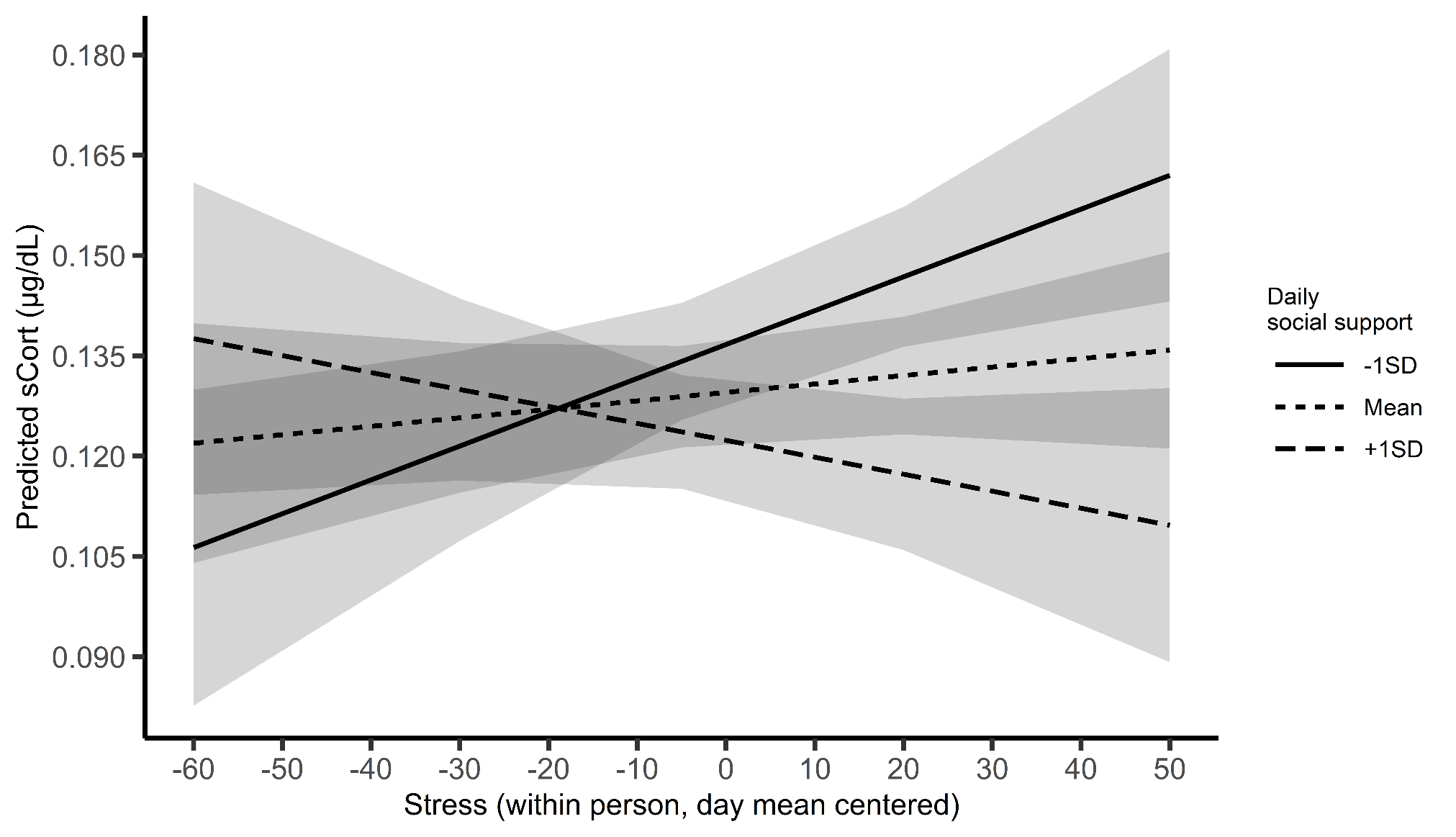
Fig. S2 Average predicted sCort as a function of the cross-over interaction of subjective stress (L1) and daily levels of social support (L2). Mean values as well as one standard deviation below and above the mean were used as grouping levels for the moderator. The ribbons indicate standard errors. Given that untransformed sCort values were used, the figure does not depict the fixed effects described in the main text.**

**
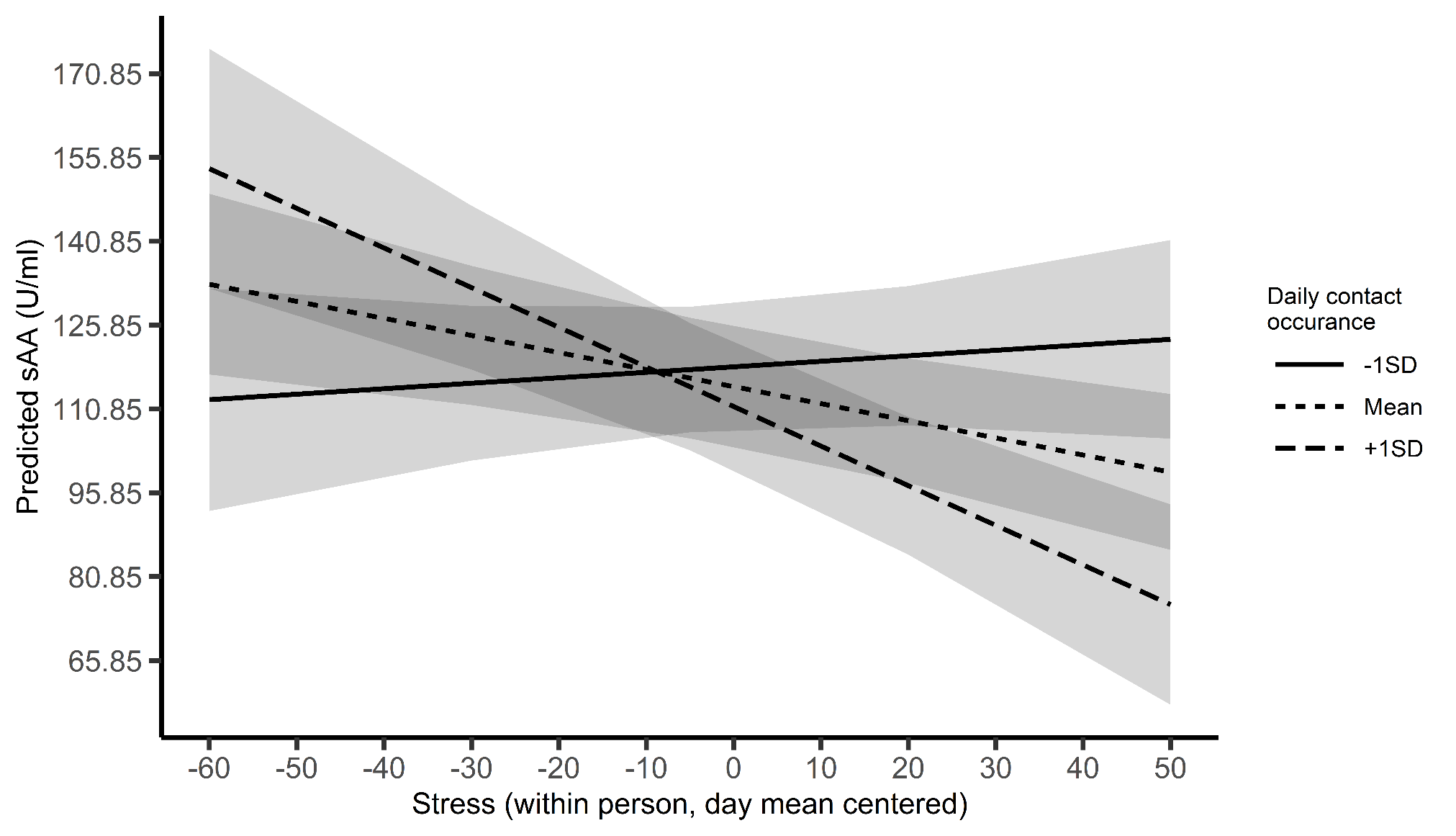
Fig. S3 Average predicted sAA as a function of the cross-over interaction effect of subjective stress (L1) and daily levels of contact occurrences (L2). Mean values as well as one standard deviation below and above the mean were used as grouping levels for the moderator. The ribbons indicate standard errors. Given that untransformed sAA values were used, the figure does not depict the fixed effects described in the main text.**
